# Carbon Nanosphere-Based TiO_2_ Double Inverse Opals

**DOI:** 10.3390/molecules30020205

**Published:** 2025-01-07

**Authors:** Dániel Attila Karajz, Kincső Virág Rottenbacher, Klára Hernádi, Imre Miklós Szilágyi

**Affiliations:** 1 Department of Inorganic and Analytical Chemistry, Faculty of Chemical Technology and Biotechnology, Budapest University of Technology and Economics, Szent Gellért tér 4, H-1111 Budapest, Hungary; karajz412@edu.bme.hu (D.A.K.);; 2 Institute of Physical Metallurgy, Metal Forming and Nanotechnology, University of Miskolc, H-3515 Miskolc, Hungary; klara.hernadi@uni-miskolc.hu

**Keywords:** inverse opal, atomic layer deposition, photocatalysis

## Abstract

Inverse opals (IOs) are intensively researched in the field of photocatalysis, since their optical properties can be fine-tuned by the initial nanosphere size and material. Another possible route for photonic crystal programming is to stack IOs with different pore sizes. Accordingly, single and double IOs were synthesized using vertical deposition and atomic layer deposition. In the case of the double IOs, the alternating use of the two preparation methods was successfully performed. Hydrothermally synthesized 326 and 458 nm carbon nanospheres were utilized to manufacture two different IOs; hence the name 326 nm and 458 nm IOs. Heat treatment removed the sacrificial template carbon nanospheres, and the as-deposited TiO_2_ crystallized upon annealing into nanocrystalline anatase form. Reflectance mode UV–visible spectroscopy showed that most IOs had photonic properties, i.e., a photonic band gap, and by the “slow” photon effect enhanced absorbance, except the 326 nm IO, even though it also had an increase in absorbance. The IOs were tested by photocatalytic degradation of Rhodamine 6-G under visible light. Photocatalytic experiments showed that the 458 nm IO was more active and the double IOs showed higher efficiency compared to monolayers, even if the less effective 326 nm IO was the top layer.

## 1. Introduction

Photonic crystal research has rapidly increased after the development of three-dimensional structure was achieved. E. Yablonovitch [1] and S. John [2] independently created the concept of a 3D photonic crystal in 1987. Later, in 1991, E. Yablonovitch [3] and his team made the first 3D photonic crystal, Yablonivite. The first synthesis attempts were made using a top-down method, which created higher-quality materials, but it was a harder, more expensive method [4]. The bottom-up methodology offered simpler synthesis routes. Inverse opals (IOs) have become a popular form of 3D photonic crystals, because of the easier preparation method and good control of the structure [5]. IO preparation starts with the choice of the building block, which is usually an oxide (e.g., silica) nanosphere, or a polymer nanoparticle, like polystyrene [6,7] or poly(methyl methacrylate), PMMA [8], or a carbon nanosphere [9,10]. An ordered structure can be achieved by taking advantage of colloidal self-assembly, but simple gravitational sedimentation is slow [11]. Numerous methods were developed to speed it up, like spin-coating [12], vertical deposition [13] and electrostatic-assisted layer-by-layer (LBL) assembly [14]. Another important aspect of opal preparation is the minimalization of cracks, which can be reduced by increasing the humidity [15], co-assembly of the template nanoparticle with a sol-gel precursor [16] or modifying the substrate surface [17]. Infiltration of the opal template can be achieved by solution-based techniques, like sol-gel [18] or gas-phase techniques like chemical vapour deposition (CVD) [19] or atomic layer deposition (ALD) [20]. Finally, the template is removed by calcination [21] or acidic etching [22].

IO materials are applicable to photocatalysis due to the possibility of infiltrating the opal structure with photocatalytic, semiconductor oxides, like TiO_2_ [23] and ZnO [24], and their unique optical properties. The periodic structure of the photonic IO gives rise to an optical band gap, called the photonic band gap [25]. The photonic band gap alone does not increase the photocatalytic properties, but the interference at the two edges of the photonic band gap, i.e., the “slow” photon effect, enhances light absorption in wavelengths where the material usually does not absorb [26]. IOs can be modified to change the optical properties and photocatalytic effectiveness. Examples are the incorporation of noble metal nanoparticles [27], doping with another non-metal [28] or metal [29] element and the infiltration of the opal template with more than one material, making a composite IO [30].

Another modification is to stack IOs with different layers. Stacking IOs with a layer of P25, a TiO_2_ IO/mesoporous P25 structure, performed better than pure P25 with the same layer thickness as the stacked structure [31]. Similarly, sandwiching multiple layers of IOs and P25 layers was carried out in the form of a P25/IO/P25/IO structure for solar energy conversion [32]. Apart from P25, nanocrystalline TiO_2_ was stacked with one and two Ios with different pore sizes. The IO layers increased the efficiency of the solar cell electrode by reflecting the light back in a specific spectral range, this can be further enhanced by stacking IOs with different photonic band gaps to increase the spectra range of reflection [33]. Stacking mesoporous and macroporous IOs can increase the photoconversion rates of solar cells, the reason being that the IO with smaller-sized pores adsorbed more dye (for sensitization) and the macroporous IO gave the adequate photonic band gap [34]. Double IOs can be utilized for photocatalytic purposes; e.g., Zulfiqar et al. [35] stacked two IOs, with pore sizes of 200 nm and 400 nm, and later decorated them with gold nanoparticles. Stacking three IO layers is also possible, which was demonstrated with smaller (522/608/756 nm) [36] and larger (300/600/1000 nm) [37] particle size differences. Three layers of IO can be made into a sandwich, making the bottom and the top layers of smaller and the middle layer of larger pore size. This can increase the photocatalytic efficiency by adjusting all three layers’ photonic band gap in a way that the “slow” photon effect is adjusted to the absorption edge of the TiO_2_.

The aim of this work is to prepare mono- and bilayer IOs from 326 and 458 nm carbon nanospheres as sacrificial opal templates using vertical deposition, filling up the structure with TiO_2_ by atomic layer deposition and removing the opal with 500 °C heat treatment, which also crystallizes the TiO_2_. Further goal is comparing the optical and photocatalytic properties of the 326 nm/458 nm and 458 nm/326 nm double IOs to each other and to the monolayered ones. Sample characterization was performed by scanning electron microscopy (SEM), energy dispersive X-ray analysis (EDX), X-ray diffraction (XRD) and UV–visible spectrophotometry. Photocatalytic tests were performed under UV and visible light sources using methylene blue as the model molecule for degradation.

## 2. Results

Prepared samples are named after the nanospheres used in their synthesis; this means that the IO made using 458 or 326 nm carbon nanosphere is named 458 IO and 326 IO, respectively. The double IOs are named as following: the bottom IO layer/top IO layer.

**Figure 1:**
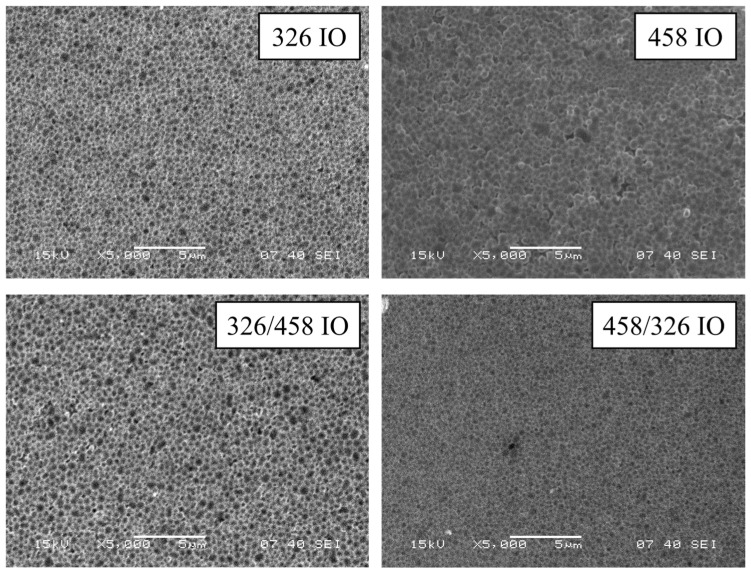
SEM images of the IO samples.

SEM images show the structure of the IO from a top view (Figure 1). The structure retained the spheroid shape of the original carbon nanospheres on the surface; also, the large deviance of nanosphere diameter is visible and makes the inverse opal less organized than conventional SiO_2_- or polystyrene-based ones. The size of the individual void spheres with the deposited TiO_2_ is larger than the initial nanosphere sizes (Figures S1–S4 and Table S1). This can be attributed to the deposition of TiO_2_ and the combination of large sphere diameter deviation. During vertical deposition, the smaller nanospheres are deposited earlier than the bigger ones; this means that the top layer of the opal will have a larger average diameter.

EDX spectroscopy provides information (Table 1) about the composition of the IOs. The samples are mostly made of Ti and O, which proves the success of ALD, but there is significant residual carbon mostly from the carbon tape. The rest of the elements, like Na, Mg, Al, Si, K, Ca and the excess O come from the glass substrate; in the case of Al, the source may be the sample holder or the inside of the SEM. The amount of Ti differs from sample to sample, and this can be understood by the different ALD layer thickness and the different height of the original opal structures.

**Figure 2:**
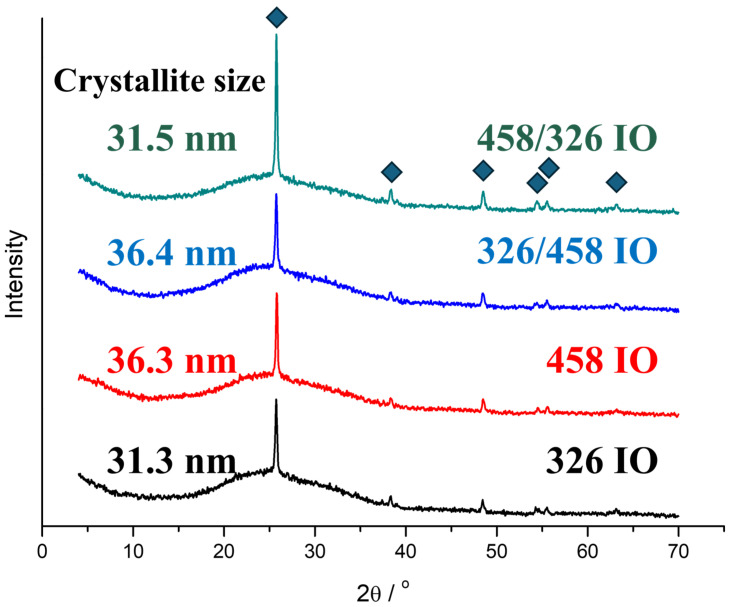
XRD diffractograms of the inverse opal samples.

The TiO_2_ IOs are crystalline and show the crystallinity reflections of anatase (Figure 2). All the IOs are identified by the ICCD card of 98-009-2363, which is nanocrystalline anatase. The only visible difference between the samples is the larger intensity of the [011] peak for the 458/326 IO. The crystallite size calculations are based on the Scherrer equation. The crystallite size depends on the ALD layer’s thickness.

**Figure 3:**
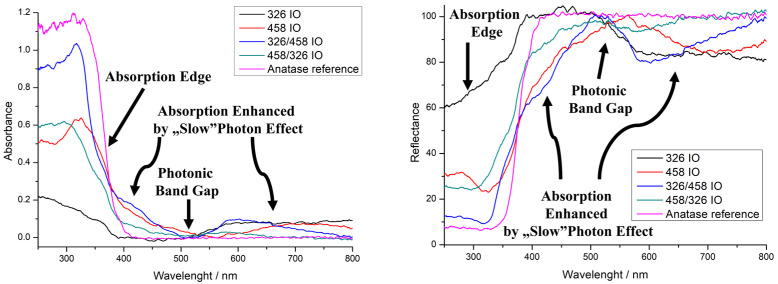
UV–vis spectra of the inverse opals (IOs), absorbance (**left**) and reflectance (**right**).

UV–visible spectroscopy was performed in reflectance mode (Figure 3). The IOs are compared to anatase powder to better represent the optical properties. All IOs show an absorption edge, which is due to the TiO_2_ semiconductor band gap. The absorption edge of the 458 IO and 326/458 IO shifted blue by 20 nm. The 326/458 IO’s absorption edge also shifted around 40 nm, while the 326 IO shows lower absorbance. The photonic band gap is one of the properties that arises from the IO structure. The 458 IO has it around 560 nm, which is close to one of our own earlier findings [10]. Double IOs also show a photonic band gap around 510 nm, while in the case of the 326 nm IO this is not that pronounced. The absorption enhancement due to the “slow” photon effect is also visible for the 458 nm IO right before and after the absorption edge at 435 nm and at 710 nm. For the double IOs, the slow photon areas are around 410 nm and 590 nm. The 326 nm IO shows an absorption increase starting from 510 nm to 1000 nm. The IO with 326 nm void size would have a photonic band gap from 400 to 500 nm. The precise enhancement of the IO’s blue and red edges depends on the structure: the order is 326/458 IO, 458 IO and 458/326 IO, where the absorbance is 0.17, 0.11 and 0.06, respectively. For the red edge, the order is 326/458 IO, 458 IO and 458/326 IO, where the absorbance is 0.10 (600 nm), 0.05 (715 nm) and 0.03 (580 nm), respectively. The 326 IO also has an increase in absorption from 510 nm, which is around 0.08.

**Figure 4:**
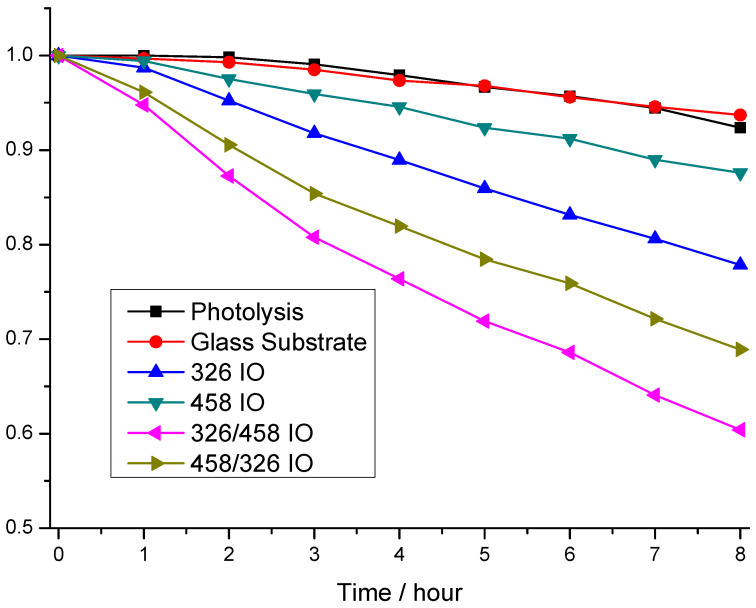
Photocatalysis experiments of the inverse opal (IO) samples, under visible light irradiation.

Rhodamine 6-G photodegradation experiments show the photocatalytic activity of the samples (Figure 4). The model pollutant photodegrades after 8 h of irradiation, but it is just around 8.0%. Interestingly, the glass substrate makes this worse (6.3%). The photocatalytic efficiency measurements show that the order of activity is 326/458 IO, 458/326 IO, 326 IO and 458 IO, where the photocatalysis degraded 39.6%, 31.0%, 22% and 12.5%. From these results, all IOs possess photocatalytic properties; moreover, the stacked IOs proved to be more efficient. Interestingly, the 326 IO shows better efficiency than the 458 IO; this can be explained by the higher specific surface area from the smaller, ~326 nm nanospheres. Also, the 326 IO has a significant absorbance in the visible region from 510 nm, which proves to be more effective than the absorption enhancement of the 458 IOs. Looking at the double IOs, the 326/458 IO proved to be more effective than the 458/326 IO, which goes against the tendency of the single IOs. The cause of this could be that the upper 458 layer provides increased absorbance, while the higher surface area is still retained, but on the opposite effect the absorption enhancement of the 458 IO layer is lost; this is visible on the UV–vis spectra of Figure 3. Interestingly, with this train of thought, the 458/326 IOs performed better compared to the 326 IO This means that the bottom 458 IO layer still improves the 326 IO’s efficiency. The UV–vis spectra show that the 458/326 IO has an increased absorbance close to the absorption edge.

The future prospects of inverse opal photocatalysis rely on its ability to be more profitable and greener. In our case, the preparation of opal crystal, the template is cheap due to the utilization of carbon nanospheres. The nanosphere preparation uses the hydrothermal method, which is cost-efficient, low-energy and green [38]. Carbon nanosphere preparation uses cheap materials, such as water, sucrose and NaOH; in the washing phase, ethanol and acetone are needed. Notably, a low temperature (70 °C) is enough. It is also pollution-free. The second step, the opal crystals infiltration, is harder. Vertical deposition is a cost-effective method, but it is really time-consuming, unless there is an additional driving force. This external impulsion can be electrical or magnetic force, which can speed it up significantly [39,40]. ALD is mainly used in the semiconductor industry due to its high precision and quality. It is possible to increase the scalability and time efficiency of ALD. Large ALD reactors are demanded in today’s world by the solar panel industry [41], while the roll-to-roll ALD method is able to continuously, “infinitely” deposit layers using a treadmill-based technique [42]. Stability of the photocatalyst can decrease by two mechanisms; it can happen either by mechanical damage or photocorrosion. While the first can be stopped by filtration, the second is harder to prevent. Thankfully, TiO_2_ photocatalysts’ big advantage is their stability, for example compared to ZnO [43] or CdS [44]. Moreover, TiO_2_ is researched as a protective layer against the photocorrosion of other photocatalysts [45,46], including inverse opals [47].

## 3. Discussion

In this work, we prepared simple (326 nm and 458 nm opal template particle size) and double IOs utilizing 326 nm and 458 nm carbon nanospheres on top of each other. The IO synthesis was achieved by vertical deposition of opals combined with atomic layer deposition of TiO_2_ and the final heat treatment, after which the IO structure was achieved. The TiO_2_ deposition was confirmed using EDX and XRD; the latter also specified the crystalline form to be anatase. Calculations using the Scherrer equation showed that the crystallite size is indeed in the nanoscale, around 31 nm and 36 nm, for the 326 IO and 458 IO, respectively. Optical properties of the IOs were compared to TiO_2_ anatase powder by reflectance mode UV–visible spectroscopy. Most IOs possessed a photonic band gap and all IOs have an absorption edge and an increasement of absorbance due to the “slow” photon effect. Photonic band gaps were observed around 560 nm, which is within 10 nm of our earlier work, and the double IOs’ photonic band gaps were closer to the band gap, at 510 nm. “Slow” photon enhancement occurred close to the absorption edge at 410–430 nm and at higher wavelengths. In the case of the 326 IO, there is an absorption increase from 510 nm. Photocatalytic studies show that all of the IOs are capable of degrading Rhodamine 6-G under visible light irradiation. The 326 IO has a better activity compared to the 458 IO, which due to a smaller initial sphere size has a higher surface area. It also has more intense visible light absorption at higher wavelengths. Double IOs have better efficiency compared to single IOs, even if the less advantageous 458 IO is on top of the better 326 IO. This synergistic behaviour shows that adding additional “slow” photon-enhanced absorbance to other regions can increase the photocatalytic efficiency and it works the other way too; an additional 326 IO layer also increases the photocatalytic activity. Now, the order of photocatalytic efficiency of the double IOs lines up with the enhancement of absorbance on the UV–vis spectra (Figure 3). Another aspect of this work is that carbon nanospheres prepared by the hydrothermal method have a significantly higher size distribution compared to SiO_2_- or polymer-based (PMMA or polystyrene) nanospheres. Despite its lower quality, it is still usable as a sacrificial template for IO photocatalysis. This can potentially open up the possibility of using lower-quality but cheaper IOs.

## 4. Materials and Methods

### 4.1. Carbon Nanospheres

Carbon nanospheres were prepared using a hydrothermal method, using sucrose solution as precursor. In the case of the large (458 nm) carbon nanospheres, the reaction ran at an alkaline pH 11 adjusted by NaOH for 12 h at 180 °C in an autoclave. Carbon nanospheres were then washed with distilled water, ethanol–water mixture and five times with acetone, in this order. The resulting powder (brown) was dried overnight at 70 °C. SEM images showed that the nanospheres had an average diameter of 458 nm with 80 nm standard deviation [10]. The small (326 nm) carbon nanospheres were prepared similarly, but with slightly different parameters. The pH of the solution was 12 (adjusted by NaOH), but it was also run for 12 h at 180 °C. The nanospheres were washed three times with distilled water, then suspended in 45% ethanol–water solution. The suspension was centrifuged at 4000 rpm for 20 min, then the settled material was filtered at 70 °C with warm distilled water overnight. SEM images showed the average diameter to be at 326 with 92 nm standard deviation.

### 4.2. Preparation of Opal Crystals

The different carbon nanospheres were used as the sacrificial templates for the opal crystals. Nanospheres were suspended in ion-exchanged water and ultrasonicated for achieving disaggregation. All carbon nanosphere suspensions had a concentration of 0.3 w%. The substrates (microscope slide) were washed with soapy water, ethanol, then distilled water, finally submerged in “piranha” solution (3:1 concentrated sulfuric acid/35% hydrogen-peroxide solution) for 1 h; the acid was washed away by ion-exchanged water. The opal crystal growth was carried out using vertical deposition. An amount of 5 mL of the suspension was added to a glass, then the substrate was put into the suspension in an angle, then it was put into an oven. The temperature program was the following: 50 °C was reached under 30 min, then it was maintained for 14 h, the temperature was raised to 80 °C under 30 min, then it was maintained for 1.5 h.

### 4.3. Atomic Layer Deposition (ALD)

IO preparation depends on the filling of the opal crystal. For this, we applied an OkyayTech Atomry T^®^ ALD from OkyayTech, Tarsus, Turkey, because its gas phase precursors can infiltrate the opal structure. TiO_2_ ALD deposition on the opal substrates used the following parameters: the pulse time for water and titanium tetraisopropoxide (TTIP) was 200 ms and the purge times between them were 30 s; the TTIP was heated up to 100 °C; the reactor was at 200 °C; the connections between them were at 150 °C; the nitrogen flow was at 10 sccm. The number of cycles depended on the pore size of the opal crystals, which was determined by the size of the base nanospheres. Accordingly, 600 and 1200 cycles were performed for the 326 and 458 nm carbon nanosphere-based opals, which resulted in a 15 and 35 nm TiO_2_ deposition. The ALD layer thickness was determined by layers deposited on reference [001] silicon wafer and measured by UV–vis reflectometry.

### 4.4. Carbon Nanosphere Removal

The final synthesis step was the removal of the sacrificial template, i.e., the carbon nanosphere-based opal crystal. Elevated temperature was used as a way to not only remove the nanospheres, but to further increase the crystallization of the TiO_2_ to anatase form. The heat program was the following: 500 °C was achieved under 4 h, then it was maintained for 2 h, then it was allowed to cool down to room temperature.

### 4.5. Single and Double IO Preparation

Double IOs were prepared by combining the methods mentioned earlier. After the preparation of the opal crystal made of 326 nm carbon nanospheres, it was coated with 15 nm of TiO_2_, but the nanospheres were not removed. Instead, another vertical deposition was performed to make a 458 nm carbon nanosphere-based opal on top of the coated opal crystal, then a 35 nm TiO_2_ coating was applied. Afterwards, then, the two (326 and 458 nm) opals were removed at the same time. The 458/326 nm IO was prepared in a reversed way, i.e., the same procedure was carried out with a 458 nm carbon nanosphere-based opal, which was coated with 35 nm TiO_2_, then a 326 nm carbon nanosphere-based opal crystal was vertically deposited on top of it, then followed by 15 nm TiO_2_ layer deposition via ALD, and then finally the two opal crystals were removed at the same time by annealing.

### 4.6. Characterization

SEM images were taken using a JEOL JSM-5500LV scanning electron microscope from JEOL, Tokyo, Japan. Samples were hold in place using adhesive carbon tape on top of a large aluminium sample holder. EDX spectra were taken with the same scanning electron microscope (SEM) device, and for each sample three EDX points were measured. Characterization of the sample’s crystallinity was carried out by a PANanalytical X’Pert Pro MPD X-ray diffractometer using Cu K-α radiation between the angle range of 5°–65°. UV–vis spectra were taken by an Avantes AvaSpec-2048 spectrophotometer equipped with optical cables from Avantes, Apeldoorn, Netherlands, using BaSO_4_ as reference for the reflective mode measurements. The graphs were baseline corrected using the anatase TiO_2_ spectrum as reference and smoothed using 20-point second-order Savitzky–Golay smoothing.

### 4.7. Photocatalysis Experiments

Photocatalytic study of the samples was carried out using Rhodamine 6-G as the model dye. The setup for the experiment was the following: the sample was attached to the wall of a large, 30 mL 3.5 × 2.2 × 5 cm quartz cuvette, 10 mL of 1.5 × 10^−5^ M solution was poured into the cuvette, a magnetic bead was added and the cuvette’s top was closed using parafilm (to decrease evaporation). The cuvette was placed between two stacks of three 18 W fluorescent lamps (visible irradiation); the stacked lamps were facing each other at 10 cm distance. Energetic T8 18W RoHS fluorescent lamps were used. Before the test, the sample was left overnight in darkness to reach adsorption equilibrium. The photocatalysis was carried out for 8 h, where every 1 h 3 mL of the solution was taken and measured with the beforementioned Avantes AvaSpec-2048 spectrophotometer, then the 3 mL was poured back to the cuvette.

## Figures and Tables

**Table 1:** EDX results of the IO samples in atom%.

Elements	326 IO	458 IO	326/458 IO	458/326 IO
C	6.0	6.6	5.3	2.8
O	65.8	61.2	64.1	66.2
Na	1.0	1.7	1.2	0.7
Mg	-	0.3	-	-
Al	0.1	0.2	-	0.1
Si	2.5	8.4	5.1	1.8
K	-	0.1	-	-
Ca	0.3	0.8	0.6	0.3
Ti	24.2	20.6	23.6	29.0

## Data Availability

The raw data supporting the conclusions of this article will be made available by the authors on request.

## Supplementary Materials

The following supporting information can be downloaded at: https://www.mdpi.com/article/10.3390/molecules30020205/s1. Figure S1. SEM image of the IO sample made with the 326 IO sample. Figure S2. SEM image of the IO sample made with the 458 IO sample. Figure S3. SEM image of the IO sample made with the 326/458 IO sample. Figure S4. SEM image of the IO sample made with the 458/326 IO sample. Table S1. Diameter of the voids, with the wall thickness.
