# Supplementary material for: Carbon Nanosphere-Based TiO2 Double Inverse Opals
Source: Molecules. 2025 Jan 7;30(2):205. doi: 10.3390/molecules30020205 (PMC11767861; doi:10.3390/molecules30020205)
Supplement: Supplementary file 1 [file molecules-30-00205-s001.zip › molecules-3392533-supplementary.pdf]

molecules

Supplementary Materials

Carbon Nanosphere-Based TiO_2 Double Inverse Opals

Dániel Attila Karajz, Kincső Virág Rottenbacher, Klára Hernádi and Imre Miklós Szilágyi

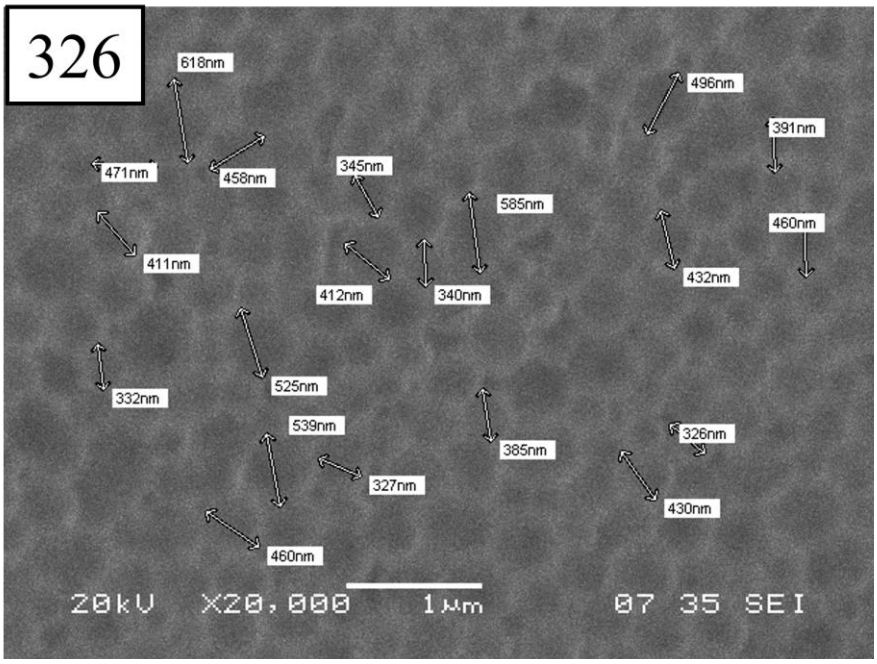

Figure S1. SEM image of the IO sample made with the 326 IO sample.

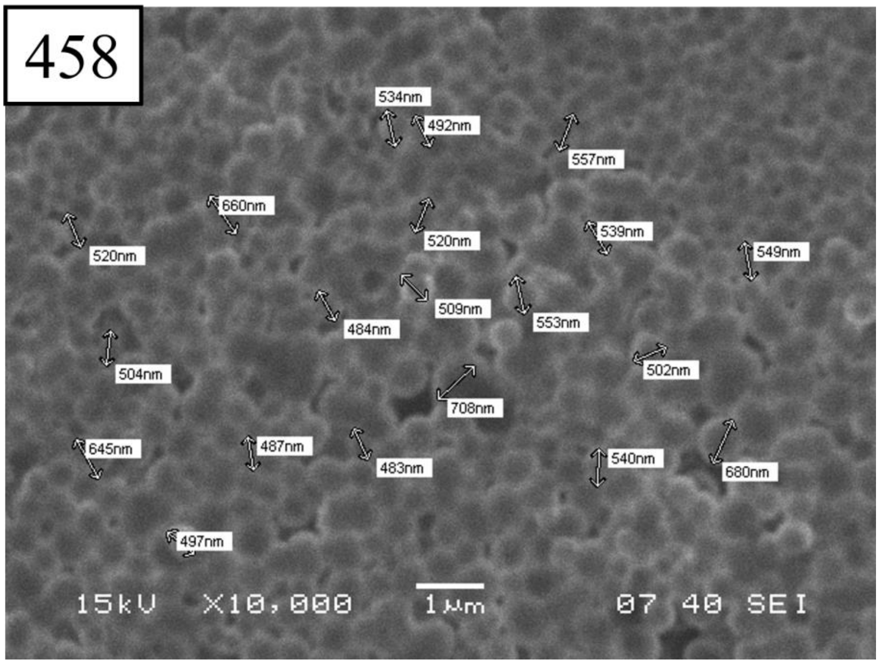

Figure S2. SEM image of the IO sample made with the 458 IO sample.

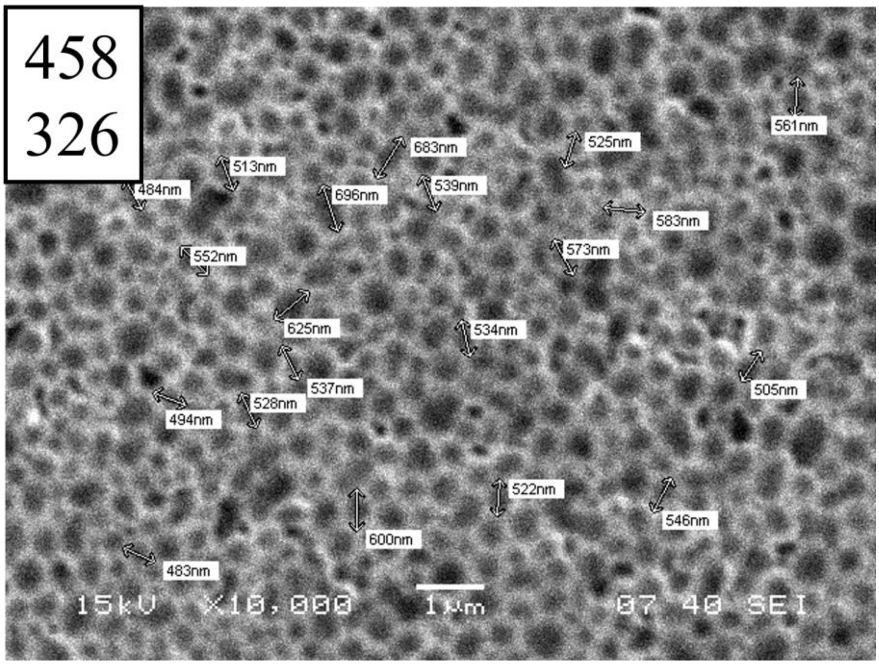

Figure S3. SEM image of the IO sample made with the 326/458 IO sample.

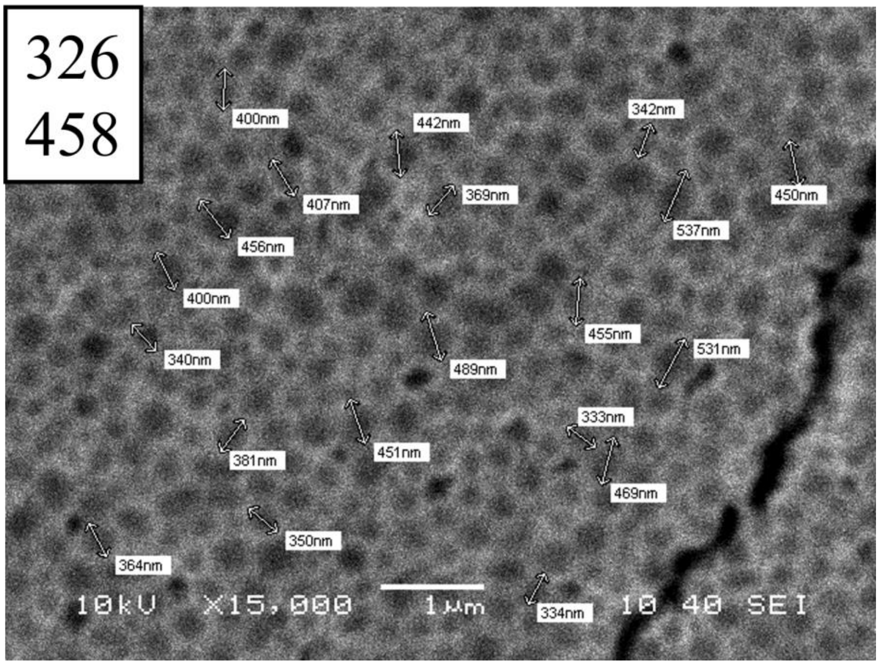

Figure S4. SEM image of the IO sample made with the 458/326 IO sample.

Table S1. Diameter of the voids, with the wall thickness.

Sample	Void diameter + wall thickness
326 IO	437 ± 85 nm
458 IO	548 ± 69 nm
326/458 IO	554 ± 59 nm
458/326 IO	415 ± 64 nm
